# Bridging the Gap: Portal Messages as a Tool to Improve Breast Cancer and Diabetes Screening Rates

**DOI:** 10.1007/s11606-026-10452-0

**Published:** 2026-04-29

**Authors:** Holly Krelle, William C. King, Sarah Tsuruo, Nathan Klapheke, Jeremy Lu, Kyra Rosen, Simon Jones, Blaire Holman, Lily Pazand, Lauren Diller, Gabriella Meringolo, Leora I. Horwitz

**Affiliations:** 1 Division of Healthcare Delivery Science, Department of Population Health, NYU Grossman School of Medicine, New York, NY USA; 2 Medical Center Information Technology, New York University Langone Health, New York, NY USA; 3 Clinically Integrated Network, NYU Langone Health, New York, NY USA

**Keywords:** patient outreach, breast cancer screening, diabetes screening, preventive care

## Abstract

**Background:**

Healthcare systems use electronic patient reminders to encourage preventive care receipt. We used an innovative randomized testing approach to identify reminder design elements associated with improved patient responsiveness.

**Aim:**

Improve patient response to electronic preventive care screening reminders.

**Setting and Participants:**

Outpatient clinics affiliated with NYU Langone Health (NYULH) and their patients. Included patients were due for retinal, hemoglobin A1c (HbA1c), or mammography screening.

**Program:**

Iterative EHR reminder redesign using a behaviorally informed framework, testing improvements based on randomized implementation. We randomized patients for each screening type to intervention (redesigned) or control (existing) reminders. Outcomes were scheduling or completion of indicated test. Plan-Do-Study-Act Cycle #1 tested all three redesigned reminders. Cycle #2 tested a personalized reminder versus the redesigned mammography reminder from Cycle #1.

**Results:**

Cycle #1: Redesigned mammography reminders showed greater adherence (25/212 [11.8%] vs. 13/212 [6.1%]) compared to controls (*p* = 0.04). Redesigned retinal and HbA1c reminders showed no difference. Cycle #2: Scheduling adherence for mammography was similar for personalized and non-personalized reminders (*p* = 0.68).

**Conclusion:**

Iterative, randomized testing of re-designed electronic preventive care reminders was feasible within a large, complex healthcare system and showed the value of brief messages with a scheduling link for mammography.

**Supplementary Information:**

The online version contains supplementary material available at 10.1007/s11606-026-10452-0.

## INTRODUCTION

Preventive care services have been covered under the Affordable Care Act since 2010.^1^ Nonetheless, a 2015 study found that only 8% of U.S. patients over age 35 received all recommended preventive care services and nearly 5% received no preventive care services at all.^2^ These services are necessary to prevent, diagnose, and/or manage chronic diseases or conditions, which are leading drivers of morbidity and healthcare costs.^3^

Given the large volume of patients not meeting screening recommendations, it is essential to develop inexpensive, scalable, and effective interventions, such as electronic patient portal reminders. Prior work by our team and others has shown that simple improvements in content and convenience of reminders can meaningfully increase uptake, and that these approaches can be developed and tested efficiently using low-cost randomized designs.^4–7^

At our large, urban academic medical center, we found that appointment scheduling rates for recommended diabetes and breast cancer tests among eligible patients were below target, even after electronic patient portal reminders highlighted screening importance. We therefore conducted several rapid, randomized quality improvement projects testing different electronic health record (EHR)-based message approaches. Here, we report on the impact of the messages on appointment rates and patient engagement.

## METHODS

### Overview

We used rapid randomized tests of reminder message variations to compare their effectiveness at encouraging patients to book appointments and complete screenings.^4^ Per the NYU Langone Quality Improvement Oversight Committee, this project met NYU Langone Health IRB criteria for quality improvement and was exempt from IRB review. Informed consent was not obtained from patients.

### Setting and Data Sources

The study took place in the outpatient clinics of NYU Langone Health (NYULH), a large, urban academic health system in New York City. The health system uses a single, integrated electronic health record (Epic, Epic Systems, Verona, WI). All data were obtained from insurance claims and the electronic health record.

### Study Population

PDSA Cycle #1 included patients due for mammogram or diabetes screening, while PDSA Cycle #2 included only patients due for mammogram. Other inclusion and exclusion criteria were the same for both cycles. Only patients enrolled in insurance plans providing regular reports of preventive service eligibility and utilization were included to ensure complete data capture. Our clinics care for approximately 260,000 patients annually covered by these 16 plans, a mix of commercial, Medicaid and Medicare payers. Mammogram (cohort 1) included females aged 50–74 years old without screening or diagnostic mammogram within two prior calendar years, excluding patients with bilateral mastectomies. Diabetes included patients aged 18–75 years old with a diabetes (type 1 or type 2) billing diagnosis and either lack of retinal eye exam within 1 year or no record of negative retinopathy within 2 years (cohort 2); or both lack of retinal eye exam and hemoglobin A1c not in control (last HbA1c > 9.0) (cohort 3). We excluded patients not enrolled in patient portal messaging.

### PDSA Cycle #1

#### Design

**Figure 1:**
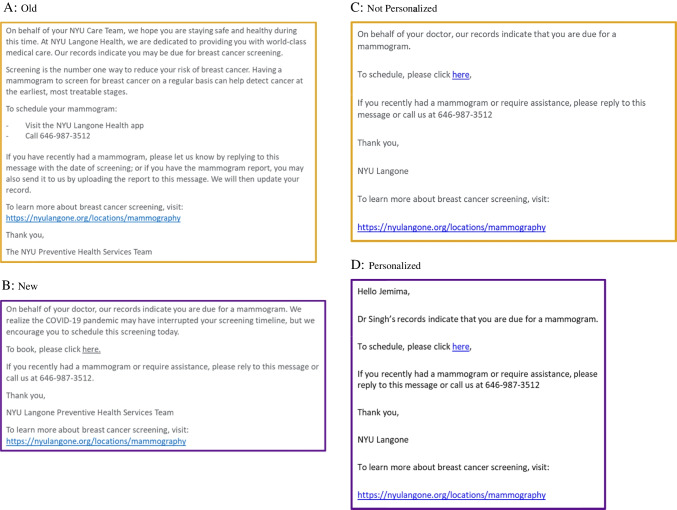
Rounds 1 and 2 mammogram messages.

Intervention messages were designed by a team including clinical and administrative outpatient leadership, communication specialists, social researchers, project associates, and analysts. The team took existing screening reminder messages and brainstormed alternatives using the EAST (Easy, Attractive, Social and Timely) behavioral science framework (Fig. S1).^8^ The final message was selected by consensus based on both evidence from other studies^9,10^ and perceived feasibility for implementation (Mammogram: Fig. 1A, B; Eye and Eye + A1c: Figs. S2 and S3). The message was designed for use within the Epic MyChart patient portal.

#### Intervention

Intervention patients received the newly designed messages, while control patients received the existing messages. Across included screening tests, intervention group patients received a shorter message than did control patients to increase *attractiveness*. Intervention group mammography messages, but not control group mammography messages, included a new direct scheduling link to increase *ease*. Both intervention and control patients received the existing direct scheduling link for the two diabetes screening tests, because it was already present in the control message.

#### Randomization and Data Collection

Within each screening test type we studied, patients were randomized 1:1 into intervention or control arms using SAS v9.4 (SAS Institute, Cary, NC). Patients received either the newly designed message (intervention) or the usual care existing message (control) via MyChart. Within each arm, patients were randomly assigned to one of eight weekly periods between July 13 and August 30, 2022, to receive their designated message to not overwhelm our call center with simultaneous appointment requests. Because the message headers for the intervention and control arms were the same, only patients who opened their message were included for each analysis to specifically study the impact of message changes.

#### Outcome Measures

The main outcome measure was the proportion of patients who scheduled their relevant screening test during the time window between opening the message and the end of data collection on September 27, 2022. We defined mammograms and retinal eye exams as scheduled based on electronic appointment records or insurance claims records within the time window. We defined HbA1c tests as scheduled based on having an appointment scheduled with primary care or endocrinology (providers who would do HbA1c testing) or a claim for a HbA1c test made within the time window. We defined the proportion of participants who scheduled either an eye or HbA1c appointment as adherent to message-based scheduling for diabetes.

#### Analysis

We compared the proportion of patients who scheduled an appointment for each type of test (mammogram, retinal exam or HbA1c) between the intervention and control groups using Pearson’s chi-squared test, with a two-sided significance cutoff of alpha = 0.05. All analyses were conducted with SAS v9.4 (SAS Institute, Cary, NC).

### PDSA Cycle #2

#### Randomization and Data Collection

The following year, we conducted a second cycle to further improve the mammogram message. We used the same protocol for selecting participants, except that, because of institutional priorities at the time, only patients eligible for mammography screening were included. Participants selected included two groups: (1) PDSA #1 patients who had not scheduled mammography appointments and (2) newly eligible patients. Patients were randomized 1:1 into intervention or control arms using SAS and randomly divided between four weekly sending periods from May 8 to May 31, 2023.

#### Intervention

Because the intervention message from PDSA #1 proved to be more effective, in PDSA #2 both intervention and control group patients received the PDSA #1 intervention base message text. Intervention patients received a new addition of the patient’s first name and the surname of their primary care doctor (Fig. 1C, D), which tested the use of personalization, to meet the EAST framework *attractive* recommendation. Including personal information such as individuals’ names has been shown to improve response rates and engagement with messages.^8,11^

#### Measures and Analysis

We used the same analytic approach and software to analyze PDSA #2 participant adherence to mammogram scheduling as we used for PDSA #1.

## RESULTS

Baseline characteristics for patients in both cycles are shown in Table S1.

### PDSA #1 Results

#### Mammogram-Eligible Patients

We randomized 371 patients to the intervention group and 371 to the control group. The rate of patients opening the message was equivalent between the intervention (212/371 or 57.1%) and control (212/371 or 57.1%); *p* = 1 (Table S2). More intervention patients (25/212 or 11.8%) compared to control patients (13/212 or 6.1%) made an appointment within the study window (*p* = 0.04) (Table 1).

**Table 1:** Proportion of Patients Who Achieved Outcome by Round

Screening message	Outcome	Control	Intervention	*p*-value
Mammogram round 1	Appointment scheduled or claim	13/212 (6.1%)	25/212 (11.8%)	0.04
Eye		22/678 (3.2%)	19/656 (2.9%)	0.71
Eye + A1C		28/195 (14.4%)	38/203 (18.7%)	0.24
Mammogram round 2		17/164 (10.4%)	15/166 (9.0%)	0.68
	Patient response	7/164 (4.3%)	17/166 (10.2%)	0.02

#### Retinal Eye Exam-Eligible Patients

A total of 978 patients were randomized to intervention and 990 to control. Similar numbers of intervention patients (656/978 or 67.1%) and control patients (678/990 or 68.5%) opened their messages (*p* = 0.50). We found no significant difference in scheduling eye exams between intervention patients (19/656 or 2.9%) and control patients (22/678 or 3.2%); *p* = 0.71 (Table 1).

#### Retinal Eye Exam and HbA1c-Eligible Patients

A total of 343 patients were randomized to intervention and 346 to control. Similar numbers of intervention patients (203/343 or 59.2%) and control patients (195/346 or 56.4%) opened their messages (*p* = 0.45). We found no significant difference in appointments between intervention patients (38/203 or 18.7%) and control patients (28/195 or 14.4%); *p* = 0.24 (Table 1). However, with this sample size, power was insufficient to detect a 5-percentage point difference.

### PDSA #2 Results

A total of 334 patients were randomized to intervention and 323 to control. Similar numbers of intervention (166/334 or 49.7%) and control patients (164/323 or 50.8%) opened their messages (*p* = 0.78) (Table S2). We found no significant difference in appointments between intervention patients (15/166 or 9.0%) compared to control (17/164 or 10.4%); *p* = 0.68 (Table 1). However, an additional post hoc outcome was noticed that showed a potential effect of personalization. Patients who received the personalized message were more likely to directly respond in MyChart (17/166 or 10.2%) than control patients (7/164 or 4.3%); *p* = 0.02 (Table 1).

## DISCUSSION

Overall, we found that shortened mammogram patient portal reminders were effective, but those for retinal eye exams and HbA1c tests were not. One reason for this might be that both new and old messages for eye/HbA1c included a direct scheduling link, whereas for mammogram, only the new message contained a link. This fits with behavioral science findings that making processes as friction-free as possible can increase response rates—for example, sending taxpayers directly to a form, rather than a webpage containing the form, increased response rates by four percentage points.^12,13^

The shortening may have had no effect. But, given no scheduling reduction in the eye/HbA1c group, this suggests it is *not worse* than a longer message—and the additional content explaining the value and importance of screening had no effect on appointment booking likelihood. Importantly, the shortened message effectiveness was sustained the following year, during PDSA #2, when both intervention and control groups generated appointment rates better than historical baseline. This provides additional reassurance that the initial effect was not false. Our findings suggest that where there are limitations to the length of messages (e.g., when using text messages), scheduling information or (potentially) personalization should be prioritized over explanatory material.

In PDSA #2, the personalized messages doubled patient engagement with clinicians via MyChart messages. However, patients who received both message types scheduled their mammogram appointments at similar rates. This suggests that personalization might help stimulate a conversation between patient and provider. Whether this is desirable likely varies by interaction—providers might want to apply personalization only when patient conversation is valuable.^8,9^ We do not have content data on messages sent to clinicians; personalized message patients may have more often replied that they had already received screening or moved elsewhere: valuable information for the practice. On the other hand, outreach about unrelated matters or simple acknowledgements might be burdensome to the practice.

The personalization round also had lower overall rates of appointment-making than the earlier round (even for the same message). This is likely because we included patients who had not made an appointment after the first round, creating a less engaged PDSA #2 population. It does leave open the chance that personalization may be effective for a more engaged population.

### Limitations

PDSA #1 included two changes to mammogram messages (shortening, scheduling link), making it difficult to disentangle the most effective element. However, no intervention effect in diabetes cohorts, which had links in both arms, suggests the link is important. PDSA #2 included both new patients and non-responders from PDSA #1, so PDSA #2 patients were likely harder to influence than PDSA #1. Lastly, while mammogram claims data were complete, appointment data were only available for the host institution. Appointment rates may therefore appear falsely low, although likely balanced given randomization.

## CONCLUSION

We used the EAST framework as the basis for redesigning and trialing EHR patient portal reminders for diabetes and breast cancer screening throughout one large healthcare system. We identified shorter length and direct scheduling links as features associated with doubled patient response to reminder messages, while personalization had no apparent effect. Different starting points, patient populations, screening types, and administrative challenges will affect which strategies work, and continued reminder message response monitoring is needed. Our work shows, however, that quality improvement trialing of electronic messages to patients—where teams send two (randomly assigned) message versions rather than one—can be a feasible, low cost, and evidence-based approach for healthcare systems to improve real time patient response to electronic screening reminders.

## Supplementary Information

Below is the link to the electronic supplementary material.

ESM 1 (131 KB DOCX)

## Data Availability

The datasets generated or analyzed during this study are not publicly available due to the presence of Protected Health Information (PHI) but de-identified data can be made available from the corresponding author on reasonable request.
