# Supplementary material for: Bridging the Gap: Portal Messages as a Tool to Improve Breast Cancer and Diabetes Screening Rates
Source: J Gen Intern Med. 2026 Apr 29;41(9):2624–8. doi: 10.1007/s11606-026-10452-0 (PMC13305094; doi:10.1007/s11606-026-10452-0)
Supplement: Supplementary file 1 — (131 KB DOCX) [file 11606_2026_10452_MOESM1_ESM.docx]

Figure S1: EAST framework used by NYU team (adapted from BIT)*^8^*

| **Easy​**  Defaults (do the pre-set option); reduce hassle (one click scheduling); simple (clear message, one action).**​**  *e.g. direct scheduling link* | **Attractive​**  Attract attention using color, images, personalization; use rewards and sanctions**​**  *e.g. “Hello [patient name], [patient’s doctor’s name]’s records indicate,”* |
| --- | --- |
| **Social**​  Use social norms (other people are doing this); leverage social networks; encourage people to make a commitment to others​  *not included in this intervention* | **Timely**​  Prompt when people are receptive (habits already disrupted); consider immediate costs and benefits; help people make (and commit to) a plan​  *e.g.*  “*we encourage you to schedule this screening today”* |

Figure S2: Round 1 Eye Exam Messages

New


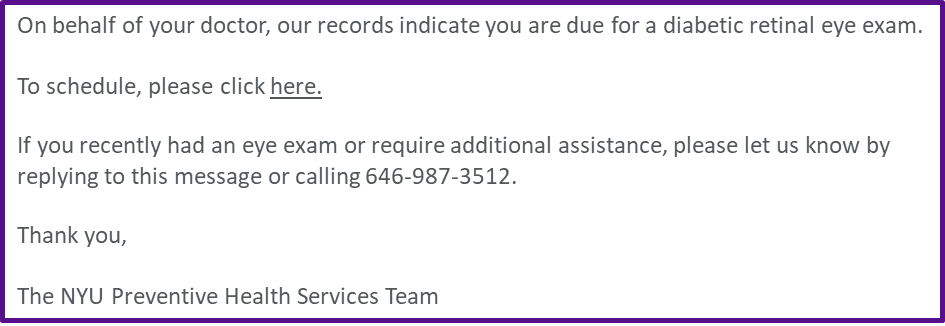


Old


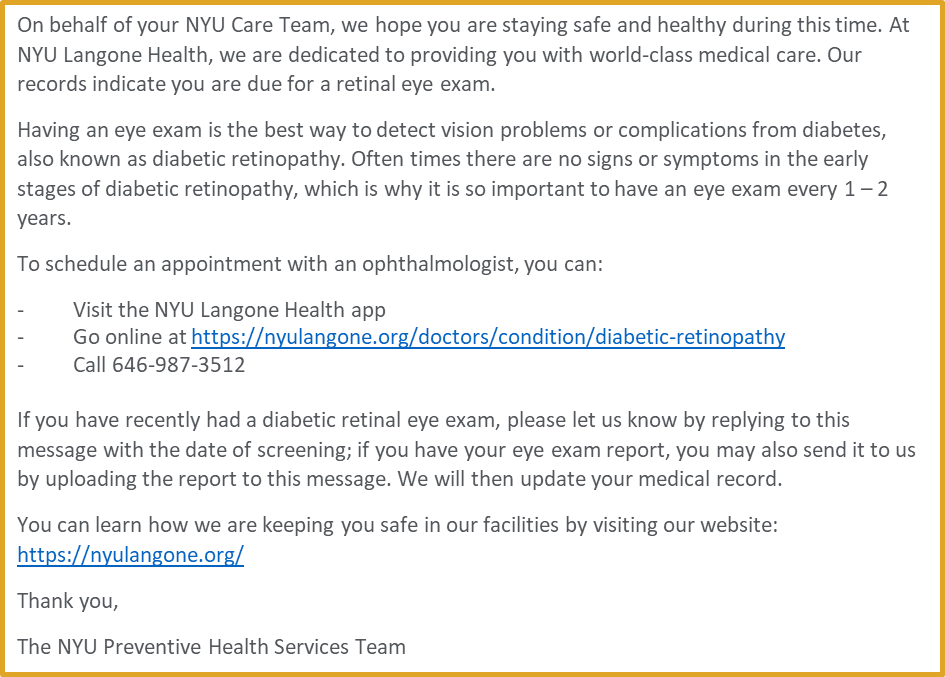


Figure S3: Round 1 Eye + A1c Exam Messages

New


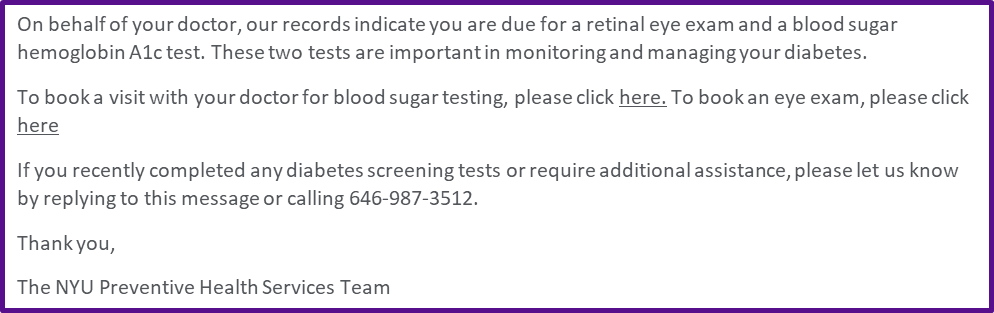


Old


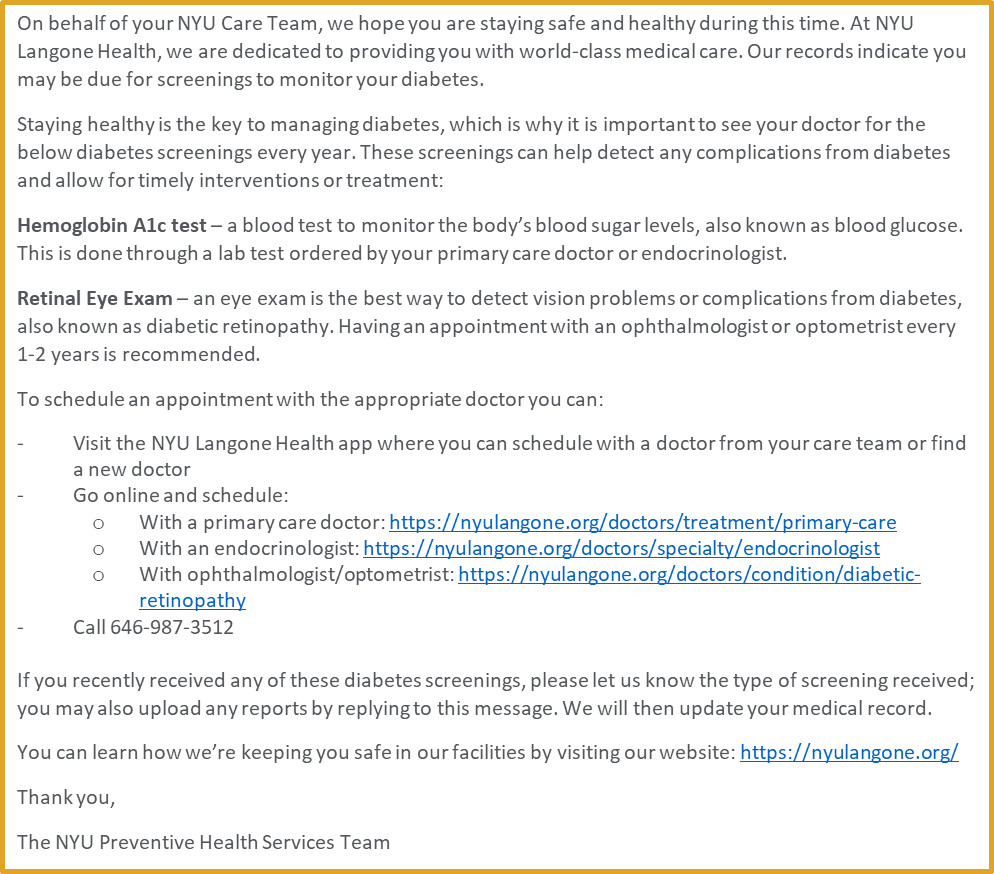


Table S1: PDSA Cycles #1 & #2 Baseline Characteristics

|  |  | |  | |  | |  | |  |  |  |  |  |
| --- | --- | --- | --- | --- | --- | --- | --- | --- | --- | --- | --- | --- | --- |
|  | | **PDSA Cycle #1** | | | | | | | | | | **PDSA Cycle #2** | |
|  |  | **Mammogram** | | | | **Eye** | | | | **Eye+Hb A1c** | | **Mammogram** | |
|  |  | **Con**  **(N=212)** | | **Int**  **(N=212)** | | **Con**  **(N=678)** | | **Int**  **(N=656)** | | **Con**  **(N=195)** | **Int**  **(N=203)** | **Con (N=164)** | **Int (N=166)** |
| Age years Median [IQR] | | 61  [51-66] | | 60  [56-65] | | 59  [52-65] | | 59  [51-65] | | 57  [49-64] | 57  [49-63] | 61.5  [56-67] | 61  [56-68] |
| Sex % F | | 100.0% | | 100.0% | | 47.0% | | 43.8% | | 43.6% | 46.3% | 100.0% | 100.0% |
| Race | |  | |  | |  | |  | |  |  |  |  |
| Asian | | 2.4% | | 6.1% | | 5.6% | | 7.2% | | 4.6% | 6.4% | 6.1% | 6.6% |
| Black | | 15.6% | | 12.7% | | 15.2% | | 14.5% | | 18.0% | 19.7% | 17.7% | 15.7% |
| White | | 63.7% | | 59.9% | | 56.9% | | 60.7% | | 58.5% | 53.2% | 50.6% | 53.0% |
| Other | | 9.9% | | 10.4% | | 11.7% | | 8.5% | | 12.3% | 12.3% | 5.5% | 7.2% |
| Unknown | | 7.6% | | 9.0% | | 7.2% | | 6.4% | | 6.7% | 7.4% | 20.1% | 17.5% |
| Missing | | 0.9% | | 1.9% | | 3.4% | | 2.7% | | 0.0% | 1.0% | 0.0% | 0.0% |
| Ethnicity | |  | |  | |  | |  | |  |  |  |  |
| Hispanic | | 12.3% | | 11.8% | | 12.1% | | 11.1% | | 13.3% | 11.8% | 11.6% | 15.7% |
| Not Hispanic | | 69.8% | | 68.9% | | 70.9% | | 70.1% | | 67.2% | 70.9% | 62.8% | 58.4% |
| Unknown | | 17.0% | | 17.5% | | 14.0% | | 15.7% | | 18.0% | 16.8% | 23.2% | 24.7% |
| Missing | | 0.9% | | 1.9% | | 3.0% | | 3.1% | | 1.5% | 0.5% | 2.4% | 1.2% |
| Insurance Type | |  | |  | |  | |  | |  |  |  |  |
| Medicaid | | 20.3% | | 19.8% | | 7.5% | | 8.2% | | 13.9% | 14.3% | 29.9% | 28.9% |
| Medicare | | 25.0% | | 22.6% | | 14.3% | | 16.5% | | 78.0% | 8.9% | 29.3% | 28.3% |
| Commercial | | 54.7% | | 57.6% | | 78.2% | | 75.3% | | 8.2% | 76.9% | 40.9% | 42.8% |
| Language | |  | |  | |  | |  | |  |  |  |  |
| English | | 96.2% | | 96.7% | | 97.9% | | 96.9% | | 93.9% | 98.5% | 92.7% | 93.4% |
| Other | | 3.8% | | 3.3% | | 2.1% | | 3.1% | | 6.2% | 1.5% | 7.3% | 6.6% |

Table S2: Message opening rates for PDSA Cycles 1 & 2 by screening type

| Screening message | Control | Intervention | p-value |
| --- | --- | --- | --- |
| Mammogram Round 1 | 212/371  (57.1%) | 212/371  (57.1%) | 1 |
| Eye | 678/990  (68.5%) | 656/978  (67.1%) | 0.50 |
| Eye + A1C | 195/346  (56.4%) | 203/343  (59.2%) | 0.45 |
| Mammogram Round 2 | 164/323  (50.8%) | 166/334  (49.7%) | 0.78 |
